# Characterizing participants who respond to text, email, phone calls, or postcards in a SARS-CoV-2 prevalence study

**DOI:** 10.1186/s12889-024-18550-6

**Published:** 2024-04-22

**Authors:** Thomas J. Duszynski, William Fadel, Brian Dixon, Constantin T. Yiannoutsos, Paul Halverson, Nir Menachemi

**Affiliations:** 1 Indiana University Richard M. Fairbanks School of Public Health, Indianapolis, IN USA; 2 Regenstrief Institute, Inc, Indianapolis, IN USA

**Keywords:** Recruitment, Cross-Sectional, SARS-CoV-2, Respondents, Text/email, Modality

## Abstract

**Introduction:**

Multiple modalities and frequencies of contact are needed to maximize recruitment in many public health surveys. The purpose of this analysis is to characterize respondents to a statewide SARS-CoV-2 testing study whose participation followed either postcard, phone outreach or electronic means of invitation. In addition, we examine how participant characteristics differ based upon the number of contacts needed to elicit participation.

**Methods:**

This is a cross-sectional analysis of survey data collected from participants who were randomly selected to represent Indiana residents and were invited to be tested for Covid-19 in April 2020. Participants received invitations via postcard, text/emails, and/or robocalls/texts based upon available contact information. The modality, and frequency of contacts, that prompted participation was determined by when the notification was sent and when the participant responded and subsequently registered to participate in the study. Chi square analyses were used to determine differences between groups and significant findings were analyzed using multinomial logistic regression.

**Results:**

Respondents included 3,658 individuals and were stratified by postcards (7.9%), text/emails (26.5%), and robocalls/text (65.7%) with 19.7% registering after 1 contact, 47.9% after 2 contacts, and 32.4% after 3 contacts encouraging participation. Females made up 54.6% of the sample and responded at a higher rate for postcards (8.2% vs. 7.5%) and text/emails (28.1 vs. 24.6%) as compared to males (χ^2^ = 7.43, *p* = 0.025). Compared to males, females responded at a higher percentage after 1 contact (21.4 vs. 17.9%, χ^2^ = 7.6, *p* = 0.023). Those over 60 years responded most often after 2 contacts (χ^2^ = 27.5, *p* < 0.001) when compared to others at younger age groups. In regression analysis, participant sex (*p* = 0.036) age (*p* = 0.005), educational attainment (p = < 0.0001), and being motivated by “free testing” (*p* = 0.036) were correlated with participation in the prevalence study.

**Discussion:**

Researchers should be aware that the modality of contact as well as the number of prompts used could influence differential participation in public health studies. Our findings can inform researchers developing studies that rely on selective participation by study subjects. We explore how to increase participation within targeted demographic groups using specific modalities and examining frequency of contact.

**Supplementary Information:**

The online version contains supplementary material available at 10.1186/s12889-024-18550-6.

## Introduction

Public health researchers need to recruit and successfully enroll participants in many epidemiological studies. Individuals make participation decisions which can be influenced by many factors including how they were contacted, the topic of study, and cultural or social contexts [1, 2]. Researchers typically use multiple methods of contact to reach potential participants which includes postcards [3], email [4], and/or text message [5]. The methods used by researchers to contact individuals to elicit participation can influence the degree to which respondents represent the population of interest [6]. There is a growing body of evidence to suggest that multiple channels of contact using a range of modalities will improve participation in research studies [7, 8].

Postcard invitations have historically been used to successfully recruit participants and can be effective when used in conjunction with contemporary electronic and web-based enrollment methods [9]. Postcards are also effective in long term studies in assuring participant retention [10–12]. Telephoning potential participants is most effective with slightly older populations because direct interaction may be favored over other electronic means by some [13]. However, many potential participants lack land line telephones or may use caller ID to block unsolicited calls resulting in lower response rates and/or nonresponse bias [14]. Electronic means of contacting potential participants, including text message and email, is increasing [15]. While text messages and emails are effective in recruiting and reducing loss to follow-up, they can be used in a wide range of settings [16, 17]. Text messages and emails can also be used to address barriers which include lack of home internet access, travel time, and recruitment while being cognizant of protecting personal health information [18, 19]. Overall, multiple methods of contact and the number of contacts, when used in combination, can increase overall response rates. However, to maximize participation and minimize effort, researchers would benefit from predictors of response based on demographics, exposure, and other motivators for study participation [6, 15].

In the context of conducting a statewide random sample SARS-CoV-2 prevalence study that used multiple methods to contact participants, the purpose of the current study is to characterize the respondents whose participation followed either the postcard, phone outreach, or electronic means of invitation. We examine how the number of outreach attempts is related to participant characteristics. Specifically, we analyze the demographic characteristics, disease symptoms and experiences, overall health status, and reasons for participation in the study among respondents of each type and number of outreach attempts. Previous research has not examined symptomology and/or health status in understanding reasons for study participation. In the current analysis, we include these variables given that our data was collected at the early stages of an infectious disease pandemic where symptoms and health status were risk factors for morbidity and mortality. The current analysis will help provide an understanding as to participant response to different modalities of contact and attempts of contacts. We believe these findings can be useful for researchers who want to maximize the benefit of outreach methods that can influence the generalizability of their study.

## Methods

The current analysis utilizes a cross-sectional study design to analyze survey data collected in April 2020 (See supplementary file) as part of a statewide population prevalence study of SARS-CoV-2 in Indiana. To better understand the data in the current study, we described the parent study [20] in which Indiana residents were randomly selected from among state residents who filed tax returns and had some contact information from other state databases. Participants who opted into the study, which took place early in the pandemic before vaccines were available, were PCR tested for active viral infection and simultaneously tested for antibodies indicating previous SARS-CoV-2 infection. Individuals who moved out of state, were less than 12 years old, were deceased, or institutionalized were excluded.

The parent study was publicly announced at a press conference with the Governor of Indiana and postcard notifications were sent to potential participants on April 24, 2020. Depending on available contact information in any state database (e.g., Bureau of Motor Vehicles, Medicaid, etc.) participants received a follow-up invitation by text/email 24 h after initial invitation, and then robocalls/texts 24 h after the initial text/emails. The robocalls/texts encouraged the selected individuals to opt into the study by registering either online or by calling a registration hotline and scheduling a date and time to get tested. All notifications and registrations were completed by close of business April 29, 2020, which resulted in 3,658 participants out of 15,495 invited [20]. The outcome of the study was participation in the prevalence study which was measured by getting tested after registering, which resulted in 99.2% of those who registered were tested.

Participants who registered for the study were tested at 68 facilities across the state and completed a short questionnaire online at time of registration, or when they arrived at a testing site, asking about health status, previous diagnoses, and reasons for participating in the study. All participants were also asked about any symptoms they were experiencing before being tested.

The main dependent variable in the current analysis is modality of contact that immediately preceded by participants’ registration for the study. If someone registered after receiving the postcard and before the text/email was sent, then they were considered as having been induced to participate by the postcard. The three groups of participants include those registering after [1] the postcard prompt [2], electronic text message and simultaneous email prompt (Text/Email); and [3] a robocall and simultaneous text message prompt (Robocall/Text). Because of variability in contact information available, not all respondents received all three methods of contact. As such, we created an additional variable measuring the unique number of initiated contacts to each participant. With this variable, we analyzed how the number of individual contacts was associated with participant characteristics.

The independent variables in our analysis were primarily from the registration survey and included demographics or other characteristics. Demographics include sex, age, race, ethnicity, and highest educational attainment. Other characteristics include reasons for participation in the study, self-reported health status, current Covid-19 symptoms, and previous exposure to Covid-19. Reasons for participation were measured on a 4-point Likert scale ranging from “not important” to “very important” and included the following items assessing potential personal, clinical, or societal benefits. Items measuring personal benefits included *“feel good contributing to Covid-19 research,” “gaining knowledge about own Covid-19 status,”* and *“testing is free of charge.”* Clinical benefits included *“less risk of transmitting Covid-19 to family and friends”* and societal benefits included *“helping inform public health officials about Covid-19,” “contributing to scientific knowledge,”* and *“receiving support from family and friends.”*

Descriptive statistics were calculated for the sample using frequencies and percentages. The analysis consisted of bivariate Chi square tests for each variable of interest by contact modality (e.g., postcard, text/email, robocall/text) and for the number of contacts for each participant. Variables that were statistically significant in bivariate analysis were included in a multinomial logistic regression to generate adjusted odds ratios and confidence intervals. All analyses were performed using SAS v.9.4 and p-values less than 0.05 were deemed statistically significant. The Institutional Review Board (IRB) at Indiana University deemed the study as not human subjects research under the public health surveillance exemption of the parent study.

## Results

Of the 15,495 invited, the study sample included 3,658 individuals including 288 (7.9%) respondents that registered for the study after receiving a postcard, 968 (26.5%) who registered after receiving a text/email, and 2,402 (65.7%) that registered after receiving a robocall/text. There were 722 (19.7%) participants that registered after 1 contact; 1,753(47.9%) who registered after 2 contacts, and 1,183 (32.4%) who registered after receiving 3 contacts. Table 1 presents the demographic characteristics of respondents which differed by modality. Females made up 54.6% of the sample and responded at a higher rate for postcards (8.2% vs. 7.5%) and text/emails (28.1 vs. 24.6%) as compared to males (χ^2^ = 7.43, *p* = 0.025). In the same crosstabulation, males responded more frequently following robocalls/text at a higher rate than females (67.9% vs. 63.7%). No significant differences by modality were observed for race.

**Table 1:** Demographic characteristics of the sample by modality

Characteristics	Postcards *n* = 288 (7.9%)	Text/Email *n* = 968 (26.5%)	Robocalls/Texts *n* = 2,402 (65.7%)	*P*-Value
Female (*n* = 1,995)	164 (8.2%)	561 (28.1%)	1,270 (63.7%)	**0.025**
Male (*n* = 1,656)	124 (7.5%)	407 (24.6%)	1,125 (67.9%)	
White (*n* = 3,373)	268 (7.9%)	889 (26.4%)	2,216 (65.7%)	0.787
Non-White (*n* = 285)	20 (7.1%)	79 (27.8%)	186 (65.3%)	
Age: <40 (*n* = 1,066)	102 (9.6%)	261 (24.5%)	703 (65.9%)	**< 0.005**
Age: 40–59 (*n* = 1,376)	118 (8.6%)	393 (28.6%)	865 (62.9%)	
Age: 60+ (*n* = 1,216)	68 (5.6%)	314 (25.8%)	834 (68.6%)	
**Education Level**				
High School or less (*n* = 935)	35 (3.7%)	211 (22.6%)	689 (73.7%)	**< 0.001**
1–3 years College (*n* = 1,046)	98 (9.4%)	255 (24.4%)	693 (66.25%)	
4 years + College (*n* = 1,631)	152 (9.3%)	499 (30.59%)	980 (60.9%)	

The age of participant was statistically different among the modalities. Specifically, those < 40 years responded at a higher rate for postcard, while those 40–59 years responded at a higher rate for text/email, and those 60 + responded at a higher rate for robocall/texts (χ^2^ = 20.1, *p* < 0.005). Education levels also differed by modality with those with the highest educational attainment responding most frequently to both postcards and text/emails than the other categories of education (χ^2^ = 61.8, *p* < 0.001). In the same crosstabulation, those with the lowest educational attainment responded at a higher rate for robocall/texts.

Differences in respondent health status and Covid-19 symptoms by modality are displayed in Table 2. Whereas there was no difference in self-reported health status by modality, significant differences by modality were observed by number of reported Covid-19 symptoms (χ^2^ = 50.1, p = < 0.001). Respondents reporting three or more symptoms responded to postcards and text/emails at the highest rates. Among those that reported no current symptoms from Covid-19, the highest percentage of participants responded after the robocalls/text notification.

**Table 2:** Health status and symptoms reported by the participant by modality

Characteristics	Postcards *n* = 288 (7.9%)	Text/Email *n* = 968 (26.5%)	Robocalls/Texts *n* = 2,402 (65.7%)	*P*-Value
Health Status				
Excellent or Very Good (*n* = 2,004)	145 (7.2%)	550 (27.5%)	1,309 (65.3%)	0.424
Good (*n* = 1,297)	114 (8.8%)	337 (25.9%)	846 (65.3%)	
Fair or Poor (*n* = 279)	23 (8.3%)	68 (24.4%)	188 (67.4%)	
**Symptoms**				
Participants reported having 0 symptoms (*n* = 2,090)	128 (6.1%)	504 (24.1%)	1,458 (69.8%)	**< 0.001**
Participant reported having 1 symptom (*n* = 553)	46 (8.3%)	157 (28.4%)	350 (63.3%)	
Participant reported having 2 symptoms (*n* = 426)	46 (10.8%)	118 (27.7%)	262 (61.5%)	
Participant reported having 3 or more symptoms (*n* = 589)	68 (11.5%)	189 (32.1%)	332 (56.4%)	

Motivations for participating in the study are displayed in Table 3. Individuals responding following the robocalls/texts indicated that *testing is free of charge* was somewhat or very important in rates higher than respondents who participated following other modalities (χ^2^ = 7.87, *p* = 0.019). No other differences in reasons for participation were observed by modality. Those that responded following the robocalls/texts reported rates of living with someone who was previously positive for Covid-19 in the household at higher rates (χ^2^ = 7.07, *p* = 0.029). No other differences in respondent positivity rates were observed.

**Table 3:** Possible motivators for participating by modality

Characteristics	Postcards *n* = 288 (7.9%)	Text/Email *n* = 968 (26.5%)	Robocalls/Texts *n* = 2,402 (65.7%)	*P*-Value
Reasons for Participating				
Feel good contributing to research Not important/barely important (*n* = 131) Somewhat/Very important (*n* = 3,527)	9 (6.9%) 279 (7.9%)	25 (19.1%) 943(26.7%)	97 (74.1%) 2,402 (65.4%)	0.104
Gaining knowledge of own Covid-19 status Not important/barely important (*n* = 231) Somewhat/Very important (*n* = 3,427)	17 (7.4%) 271 (7.9%)	48 (20.8%) 920 (26.9%)	166 (71.9%) 2,236 (65.3%)	0.276
Testing is free of charge Not important/barely important (*n* = 782) Somewhat/Very important (*n* = 2,876)	66 (8.5%) 222 (7.7%)	235(30.1%) 733 (25.5%)	481 (61.5%) 1,921 (66.8%)	**0.019**
**Covid-19 Positivity**				
PCR Positive (*n* = 47)	5 (1.7%)	10 (1.0%)	32 (1.3%)	0.610
Antibody Positive (*n* = 54)	4 (1.4%)	12(1.2%)	38 (1.6%)	0.837
Any positivity (PCR or Antibody) (*n* = 85*)	8 (2.8%)	17 (1.8%)	60 (2.5%)	0.376
**Household Exposure**				
Someone in household was previously positive for Covid-19 (*n* = 52)	3 (5.8%)	6 (11.5%)	43 (82.7%)	**0.029**

* Any positive includes both PCR and Antibody test and persons could have been both PCR and Antibody positive

The number of contacts that each participant received before registering for the study is displayed in Table 4. Compared to males, females responded at a higher percentage after 1 contact (21.4 vs. 17.9%, χ^2^ = 7.6, *p* = 0.023). With respect to age, those 60 + years of age responded by registering most often after 2 contacts (χ^2^ = 27.5, *p* < 0.001). The relationship between educational attainment and modality of response was also statistically significant. Specifically, those with the highest educational attainment, responded most frequently after 1 contact, while those with the lowest educational attainment responded after either 2 or 3 contacts most frequently (χ^2^ = 63.7, *p* < 0.001).

**Table 4:** Number of contacts by demographic and characteristics of participants

	1 contact *n* = 722 (19.7%)	2 contacts *n* = 1,753 (47.9%)	3 contacts *n* = 1,183 (32.4%)	*P*-value
Characteristic				
Female (*n* = 1,995)	426 (21.4%)	949 (47.6%)	620 (31.1%)	**0.023**
Male (*n* = 1,656)	296 (17.9%)	802 (48.4%)	558 (33.7%)	
White Persons ( *n* = 3,373)	671 (19.9%)	1,607 (47.6%)	1,095 (32.5%)	0.486
Non-White Persons ( *n* = 285)	51 (17.9%)	146 (51.3%)	88 (30.9%)	
Age: <40 (*n* = 1,066)	230 (21.6%)	471 (44.2%)	365 (34.3%)	**< 0.001**
Age: 40–59 (*n* = 1,376)	305 (22.2%)	656 (47.7%)	415 (30.2%)	
Age: 60+ (*n* = 1,216)	187 (15.4%)	626 (51.5%)	403 (33.2%)	
**Education Level**				
High School or less (*n* = 935)	113 (12.1%)	465 (49.7%)	357 (38.2%)	**< 0.001**
1–3 years College (*n* = 1,046)	209 (19.9%)	495 (47.3%)	342 (32.7%)	
4 years + College (*n* = 1,631)	395 (24.2%)	777 (47.6%)	459 (28.2%)	
**Health Status**				
Excellent or Very Good (*n* = 2,004)	381 (19.1%)	984 (49.1%)	639 (31.9%)	0.512
Good (*n* = 1,297)	270 (20.8%)	614 (47.4%)	413 (31.8%)	
Fair or Poor (*n* = 320)	65 (20.3%)	144 (45.0%)	111 (34.7%)	
**Symptoms**				
Participants reported having 0 symptoms (*n* = 2,090)	412 (19.7%)	975 (46.7%)	703 (33.6%)	0.191
Participant reported having 1 symptom (*n* = 553)	103 (18.6%)	269 (48.6%)	181 (32.7%)	
Participant reported having 2 symptoms (*n* = 426)	91 (21.4%)	201 (47.2%)	134 (31.5%)	
Participant reported having 3 or more symptoms (*n* = 589)	116 (19.7%)	308 (52.3%)	165 (28.1%)	
**Reasons for Participating**				
Feel good contributing to research Not important/barely important (*n* = 131) Somewhat/Very important (*n* = 3,527)	20 (15.3%) 702 (19.9%)	51 (38.9%) 1,702 (48.3%)	60 (45.8%) 1,123 (31.9%)	**0.003**
Gaining knowledge of own Covid-19 status Not important/barely important (*n* = 231) Somewhat/Very important (*n* = 3,427)	38 (16.5%) 684 (19.9%)	101 (43.7%) 1,652 (48.2%)	92 (39.8%) 1,091 (31.8%)	**0.038**
Testing is free of charge Not important/barely important (*n* = 782) Somewhat/Very important (*n* = 2,876)	167 (21.4%) 555 (19.3%)	397 (50.8%) 1,356 (47.2%)	218 (27.9%) 965 (33.6%)	**0.011**
**Covid-19 Positivity**				
PCR Positive (*n* = 37)	8 (21.6%)	17 (45.9%)	12 (32.4%)	0.952
Antibody Positive (*n* = 54)	9 (16.7%)	25 (46.3%)	20 (37.1%)	0.717
Any positivity (PCR or Antibody) (*n* = 85)	15 (17.6%)	40 (47.1%)	30 (35.3%)	0.802
**Household Exposure**				
Someone in household was previously positive for Covid-19 (*n* = 52)	6 (11.5%)	21 (40.4%)	25 (48.1%)	**0.039**

When examining reasons for participation in the study, we observed a different rate of response by the number of contacts a participant received. Respondents that reported that *feeling good contributing to research* (χ^2^ = 11.3, *p* = 0.003) or *gaining knowledge of their own Covid-19 status* (χ^2^ = 6.5, *p* = 0.038) as somewhat or very important responded more frequently after 1 or 2 contacts. On the other hand, those that ranked either item as not important or barely important responded at the highest rates following 3 contacts. However, respondents that indicated that *testing was free of charge* was somewhat or very important responded most frequently after 3 contacts (χ^2^ = 9.2, *p* = 0.011). Lastly, participants who reported living with someone who was previously positive for Covid-19 in their household, most frequently respondent after 3 contacts (χ^2^ = 6.5, *p* = 0.039).

When analyzing the statistically significant independent variables in the logistic regression model, only sex, age, educational attainment, and a response that *free testing* was important were significantly related to modality the prompted response (see Table 5). Specifically, males were more likely than females to respond to text/emails as opposed to robocalls/texts (OR = 1.22, 95%CI 1.04–1.42). Compared to the youngest age group, those 60 years or older were less likely to respond to text/emails (OR = 0.94 95%CI 0.77–1.14) while more likely to respond to post cards (OR = 1.73, 95%CI 1.25–2.39). Compared to those with only a high school education, those with some college participated following post cards (OR = 0.37, 95%CI 0.25–0.55) less frequently than following robocalls/texts. Those with a college degree or higher responded to post cards (OR = 0.35, 95%CI 0.24–0.52) or text/emails (OR = 0.64, 95%CI 0.53–0.77) less frequently than to robocalls/texts.

**Table 5:** Multinomial logistic regression of modality of contact before registering (95% confidence intervals)

Characteristics*	Reference: Robocalls/texts	Postcards	Text/Emails	P-Value
**Female**	--	--	**--**	**0.036**
**Male**	1.0	1.09 (0.84–1.44)	**1.22 (1.04–1.42)**	
**Age**				
Age: <40	--	--	--	**0.005**
Age: 40–59	1.0	1.16 (0.86–1.55)	0.869 (0.72–1.1)	
Age: 60+		**1.73 (1.25–2.39)**	**0.94 (0.77–1.14)**	
**Education**				
High School or less	**--**	**--**	**--**	**< 0.0001**
1–3 years College	1.0	**0.37 (0.25–0.55)**	0.86 (0.69–1.07**)**	
4 years + College	1.0	**0.35 (0.24–0.52)**	**0.64 (0.53–0.77)**	
**Symptoms**				
Participants reported having 0 symptoms	**--**	**--**	**--**	0.308
Participant reported having 1 symptom	1.0	1.40 (0.96–2.05)	1.15 (0.92–1.43)	
Participant reported having 2 symptoms	1.0	1.14 (0.77–1.7)	1.03 (0.81–1.31)	
Participant reported having 3 or more symptoms	1.0	1.49 (1.02–2.17)	1.073 (0.87–1.33)	
**Free Testing: Not Important**	--	--	**--**	**0.036**
**Free Testing: Important**	1.0	1.01 (0.82–1.49)	**1.27 (1.06–1.53)**	
**No Previously Positive Household Member**	--	--	--	0.334
**Previously Positive Household Member**	1.0	1.98 (0.26–15.2)	0.42 (0.13–1.44)	

* Characteristics that were statistically significant in the chi sq analysis were fitted in the regression model

Among those who had experienced any symptoms, motivations for participating were not significant in the model (*p* = 0.308). Individuals who indicated that *free testing* was an important motivator for participation were more likely to respond to text/emails, as compared to robocalls/texts (OR 1.27, 95%CI 1.06–1.53). No other motivating reason for participation was significant in the regression model.

Table 6 presents results from the regression analysis that examined the relationship between number of contacts and each of the independent variables that were statistically significant in bivariate analysis. Compared to the youngest age group, those 60 years and older were more likely to respond after 1 contact attempt (OR = 1.35, 95%CI 1.06–1.72). In the same model, compared to those with only a high school education, those with a college degree or higher were more likely to respond after a second contact (OR = 1.24, 95%CI 1.04–1.49) and less likely to respond after 1 contact (OR = 0.55 95%CI 0.42–0.72). Lastly, individuals who indicated that receiving *free testing* was a motivating reason to participate in the study, were more likely to respond after 1 contact (OR = 1.37, 95%CI 1.08–1.75).

**Table 6:** Multinomial logistic regression of number of contacts before registering (95% confidence intervals)

Characteristics*	Reference: 3 contacts	1 Contact	2 Contacts	*P* value
**Female**	--	**--**	--	0.098
**Male**	1.0	**1.23 (1.02–1.49)**	0.94 (0.81–1.09)	
**Age**				
Age: <40	**--**	--	**--**	**0.001**
Age: 40–59	1.0	0.98 (0.78–1.23)	1.12 (0.92–1.35)	
Age: 60+	1.0	**1.35 (1.06–1.72)**	1.12 (0.96–1.41)	
**Education**				
High School or less	**--**	**--**	**--**	**< 0.0001**
1–3 years College	1.0	**0.55 (0.42–0.72)**	1.08 (0.89–1.32)	
4 years + College	1.0	**0.41 (0.32–0.52)**	**1.24 (1.04–1.49)**	
**Reasons for Participating**				
Feel good contributing to scientific research	1.0	**0.33 (0.13–0.88)**	1.58 (0.87–2.85)	0.061
Gaining knowledge of own Covid-19 status	1.0	0.67 (0.44–1.05)	1.28 (0.92–1.79)	0.155
**Free Testing: Not Important**	--	**--**	**--**	**0.003**
**Free Testing: Important**	1.0	**1.37 (1.08–1.75)**	**0.72 (0.58–0.87)**	
**No Previously Positive Household Member**	--	--	--	0.554
**Previously Positive Household Member**	1.0	2.20 (0.46–10.5)	1.04 (0.42–2.56)	

*Characteristics that were statistically significant in the chi sq analysis were fitted in the regression model

## Discussion

Successfully recruiting and enrolling participants in epidemiological studies is influenced by several factors, including how individuals are contacted, how often they are contacted, and other factors including cultural issues and demographic characteristics. In our regression analyses, males were more likely than females to respond following text/emails, as opposed to robocalls/texts. This nuance builds upon previous studies that characterize females as more likely to participate overall in health-related surveys [21, 22] by suggesting ways to increase male participation. Researchers wanting to assure adequate male representation in public health surveys may need to ensure that sufficient modalities are used, as opposed to just increased number of contacts.

In our study, those older than 60 years of age were more likely to respond following the postcard as compared robocalls/texts and were more likely to respond after only one contact. Given that advanced age was considered a significant risk factor for Covid-related morbidity and mortality, this finding is consistent with the context in which our data was collected. This oldest age group was also less likely to respond following text/email invitations relative to their youngest counterparts. Importantly, those in the youngest age group (less than 40 years) were also comprised of children aged 12–18 years of age whose email contact info on record with the state was likely that of their parents or guardians. Researchers who want to reach younger populations may need to do so through parental contact info if appropriate to the study’s aims, research design, and data availability. Previous studies have reported that those under 40 may be more difficult to recruit without multiple prompts [23]. This is consistent with our findings where the oldest age group was more likely than the youngest age group to respond after one invitation.

In our regression analyses, higher education attainment was associated with a greater likelihood of participation following robocalls/texts. In addition, higher levels of education were typically associated with needing two contacts, as opposed to three, to elicit participation. It is possible that those with higher education may have been in jobs that allowed more flexible and remote work schedules [24], thus enabling the ability to respond and participate sooner than those that participated following three prompts. Moreover, higher educational attainment may be associated with overall better health, health literacy, and awareness of risk from Covid-19 [25] which could have contributed to a less delayed participation in our study. Lastly, higher education may have been correlated with trust in governmental public health and research thus requiring slightly less prompting to participate [26].

Symptomology and previous experience with Covid-19 in the household was not associated with neither modality of invitation nor number of contacts. However, those indicating being motivated by “free testing” responded more frequently following texts/emails and following only one contact. Our data was collected at a time when access to Covid-19 tests were extremely limited in supply and were only available in emergency departments and inpatient settings. Having free access to both PCR and antibody tests was a motivator that appears to have induced earlier participation among many. This is important to note because while random selection attempts to achieve representative samples, our study highlights that participant-specific motivators still influence study engagement and is still a source of selection bias. Our findings can be put in the context of previous research that has reported how perceived exposure impacts the decision to get tested for disease associated research [27, 28]. Notably, our team has formally assessed non-response bias in previous analyses and reported minimal to none detected nonresponse bias using formal techniques [29].

Despite the new knowledge provided by our analysis, several limitations are worth noting. Findings should be interpreted within the context of the parent study which took place during the acute phase of an infectious disease pandemic. Moreover, the cross-sectional study design utilized in the current paper can only identify associations recognizing that our study is not suited for determining cause-and-effect. We recognize that of those who registered, they self-selected to participate in the study and were limited to those 12 years of age and older as well as non-institutionalized individuals in the population. These factors limit the generalizability of our findings. It should also be noted that based on contact information availability, not every participant received every modality of invitation to participate. There was a limitation in having to assign modality of participation based upon the timing of each invitation and when the individual agreed to participate in the study by registering. We acknowledge that, while helpful, this approach could have misclassified individuals who responded due to one modality and have not yet seen or were aware of a follow up modality (e.g., did not yet check email). Lastly, our study is limited by the inability to separate the effects of dual use of simultaneous modalities such as text/emails and robocall/texts.

In conclusion, recruiting and enrolling participants in primary epidemiological research is essential for public health research. Researchers can use multiple modalities and frequencies of contact to assure sufficient participation from diverse groups in their study. Our study highlighted how demographic and other characteristics are associated with participation following either postcard, text/email, or robocall/text outreach. Knowing who is most likely to respond following different contact approaches can help public health researchers assure that their study is not compromised by insufficient participation or poor external validity. This will aid researchers by allowing them to determine which modality of contact (postcards, texts, emails, robocalls) and the number of contacts that have the highest correlation of enrollment in research studies by demographic or other motivating characteristics.

### Electronic supplementary material

Below is the link to the electronic supplementary material.

Supplementary Material 1

## Data Availability

The datasets used and/or analyzed during the current study are available from the corresponding author on reasonable request.
