# Supplementary material for: Characterizing participants who respond to text, email, phone calls, or postcards in a SARS-CoV-2 prevalence study
Source: BMC Public Health. 2024 Apr 22;24:1113. doi: 10.1186/s12889-024-18550-6 (PMC11036578; doi:10.1186/s12889-024-18550-6)
Supplement: Supplementary file 1 — Supplementary Material 1 [file 12889_2024_18550_MOESM1_ESM.docx]

Questions to ask people undergoing random sample testing

**Symptoms**

Has a doctor ever told you that you are positive for COVID-19? YES NO

Has anyone currently living in your household been told by a doctor that they are positive for COVID-19? YES NO

Within the past two weeks (14 days), have you had any of the following symptoms?

- Fever YES NO
- Cough YES NO
- Shortness of breath YES NO
- Chest pain YES NO
- Muscle ache YES NO
- Chills YES NO
- Tiredness or fatigue YES NO
- Sore throat YES NO
- Runny nose YES NO
- Headache YES NO
- Diarrhea YES NO
- Vomiting YES NO
- Loss of sense of smell YES NO
- Loss of sense of taste YES NO

Did any of these symptoms require you to seek medical attention (in the past 7 days)? YES NO

**Health Status/ Health Behaviors**

In general, would you say your health is: Excellent Very good Good Fair Poor

Do you now smoke cigarettes every day, some days, or not at all?
 1 - Every day
 2- Some days
 3- Not at all

Do you currently use chewing tobacco, snuff, or snus every day, some days, or not at all?

1 - Every day
 2- Some days
 3- Not at all

Do you currently use vaping products (like Juul or e-cigarettes) every day, some days, or not at all?

1 - Every day
 2- Some days
 3- Not at all

**Reasons for Participation Today**

Please rate the importance of how each of the following potential benefits affect your willingness to participate in today’s study:

[Scale: Don’t know/Not applicable, Not important, Barely important, Somewhat important, Very important]

Potential personal ‘benefits’

1. Feel good contributing to COVID-19 research
2. Gaining knowledge about own COVID-19 status
3. Testing is free of charge

Potential personal clinical benefit

1. Less risk transmitting COVID-19 to family and friends

Potential social benefit

1. Helping to inform public health officials about COVID-19
2. Contributing to scientific knowledge
3. Receiving support from family and friends

**Demographics**

What is your Date of Birth?

What is the highest grade or year of school you completed?

1. Never attended school or only attended kindergarten
2. Grades 1 through 8 (Elementary)
3. Grades 9 through 11 (Some high school)
4. Grade 12 or GED (High school graduate)
5. 5 College 1 year to 3 years (Some college or technical school)
6. College 4 years or more (College graduate)

How many children less than 18 years of age live in your household? __________# of children

What is your race:

- Asian including Asian Indian
- Black or African American
- White
- Bi-racial or some other race not listed above

Are you of Hispanic, Latino or Spanish origin? YES NO

What is your legal sex: MALE FEMALE

If FEMALE, to your knowledge, are you now pregnant? YES NO
